# Sulforaphane Protects the Liver against CdSe Quantum Dot-Induced Cytotoxicity

**DOI:** 10.1371/journal.pone.0138771

**Published:** 2015-09-24

**Authors:** Wei Wang, Yan He, Guodong Yu, Baolong Li, Darren W. Sexton, Thomas Wileman, Alexandra A. Roberts, Chris J. Hamilton, Ruoxi Liu, Yimin Chao, Yujuan Shan, Yongping Bao

**Affiliations:** 1 Norwich Medical School, University of East Anglia, Norwich, Norfolk, United Kingdom; 2 Department of Pathology, Harbin Medical University, Harbin, Heilongjiang, P. R. China; 3 Center of Safety Evaluation of Drugs, Heilongjiang University of Chinese Medicine, Harbin, Heilongjiang, P. R. China; 4 School of Pharmacy, University of East Anglia, Norwich, Norfolk, United Kingdom; 5 School of Chemistry, University of East Anglia, Norwich, Norfolk, United Kingdom; 6 School of Food Science and Engineering, Harbin Institute of Technology, Harbin, Heilongjiang, P. R. China; Swedish Neuroscience Institute, UNITED STATES

## Abstract

The potential cytotoxicity of cadmium selenide (CdSe) quantum dots (QDs) presents a barrier to their use in biomedical imaging or as diagnostic and therapeutic agents. Sulforaphane (SFN) is a chemoprotective compound derived from cruciferous vegetables which can up-regulate antioxidant enzymes and induce apoptosis and autophagy. This study reports the effects of SFN on CdSe QD-induced cytotoxicity in immortalised human hepatocytes and in the livers of mice. CdSe QDs induced dose-dependent cell death in hepatocytes with an IC_50_ = 20.4 μM. Pre-treatment with SFN (5 μM) increased cell viability in response to CdSe QDs (20 μM) from 49.5 to 89.3%. SFN induced a pro-oxidant effect characterized by depletion of intracellular reduced glutathione during short term exposure (3–6 h), followed by up-regulation of antioxidant enzymes and glutathione levels at 24 h. SFN also caused Nrf2 translocation into the nucleus, up-regulation of antioxidant enzymes and autophagy. siRNA knockdown of Nrf2 suggests that the Nrf2 pathway plays a role in the protection against CdSe QD-induced cell death. Wortmannin inhibition of SFN-induced autophagy significantly suppressed the protective effect of SFN on CdSe QD-induced cell death. Moreover, the role of autophagy in SFN protection against CdSe QD-induced cell death was confirmed using mouse embryonic fibroblasts lacking ATG5. CdSe QDs caused significant liver damage in mice, and this was decreased by SFN treatment. In conclusion, SFN attenuated the cytotoxicity of CdSe QDs in both human hepatocytes and in the mouse liver, and this protection was associated with the induction of Nrf2 pathway and autophagy.

## Introduction

Synthesis of nanoparticles (NPs) has become increasingly common, with some NPs now being produced commercially, including cadmium selenium (CdSe) quantum dots (QDs) [1]. QDs (smaller NPs with diameter <10 nm) are becoming prominent in the biomedical field for applications in disease diagnostics, cellular and molecular tracking, end-point assay measurements, small animal imaging, therapeutic drug delivery [2] and as novel non-viral gene delivery vectors for gene silencing [3]. A recent study even suggested that CdSe QDs have great potential for the treatment of cancer using photothermal therapy [4]. However, many studies have also documented the toxicity of QDs to mammalian cells [5], and to the liver, which was found to be a major site for CdSe NP accumulation in animals [6]. Due to the potential release of cadmium ion from CdSe [7], it is important to study the effect on the liver since Cd is a known hepatotoxicant [8]. Although the mechanism of CdSe-induced cytotoxicity is not fully understood, the generation of reactive oxygen species (ROS) and oxidative damage have been implicated [1].

Chemoprevention with natural compounds represents an attractive approach to increase cellular defence against environmental and endogenous insults [9]. It has been shown that glucosinolate-derived isothiocyanates (ITCs) from cruciferous vegetables are potent inducers of phase II antioxidant/detoxification enzymes, cell cycle arrest and apoptosis [10–12]. Sulforaphane (SFN) is an extensively studied ITC that is derived from glucoraphanin under the action of the endogenous enzyme, myrosinase [13]. After absorption into cells, SFN undergoes conjugation to glutathione (GSH), a reaction catalysed by glutathione transferases (GSTs). This reaction is a driving force for SFN accumulation and reduces GSH levels in cells, resulting in the generation of intracellular stress and subsequent activation of various signalling pathways including kelch-like ECH-associated protein 1 (Keap1)-nuclear factor-erythroid 2-related factor 2 (Nrf2) [14–16]. Moreover, SFN possesses a plethora of multi-targeted effects on cells including kinases, transcriptional factors, transporters, receptors [17–22], histone deacetylases and microtubulins [23, 24]. SFN is also able to induce autophagy characterized by the formation of autophagosomes [25]. However, it is not known whether SFN can protect against CdSe QD-induced cytotoxicity in liver and/or hepatocytes, although one report suggested that activation of Nrf2 prevented cadmium-induced acute liver injury in mice [26]. There is only one report on the protective effects of dietary ITCs on the toxicity of NPs, which indicated that SFN protects against copper oxide (CuO) NPs in mouse embryonic fibroblasts (MEF) [27]. It has previously been shown that immortalised human hepatocytes are an excellent model to study SFN and the expression of Nrf2-driven antioxidant enzymes [28]. The objectives of the present study were to (i) investigate if SFN could protect CdSe QD-induced liver damage in mice; and (ii) investigate the potential protective mechanisms of SFN against CdSe QD cytotoxicity in immortalised human hepatocytes.

## Results

### Effect of SFN pre-treatment on cytotoxicity in HHL-5 cells exposed to CdSe QDs

**Fig 1:**
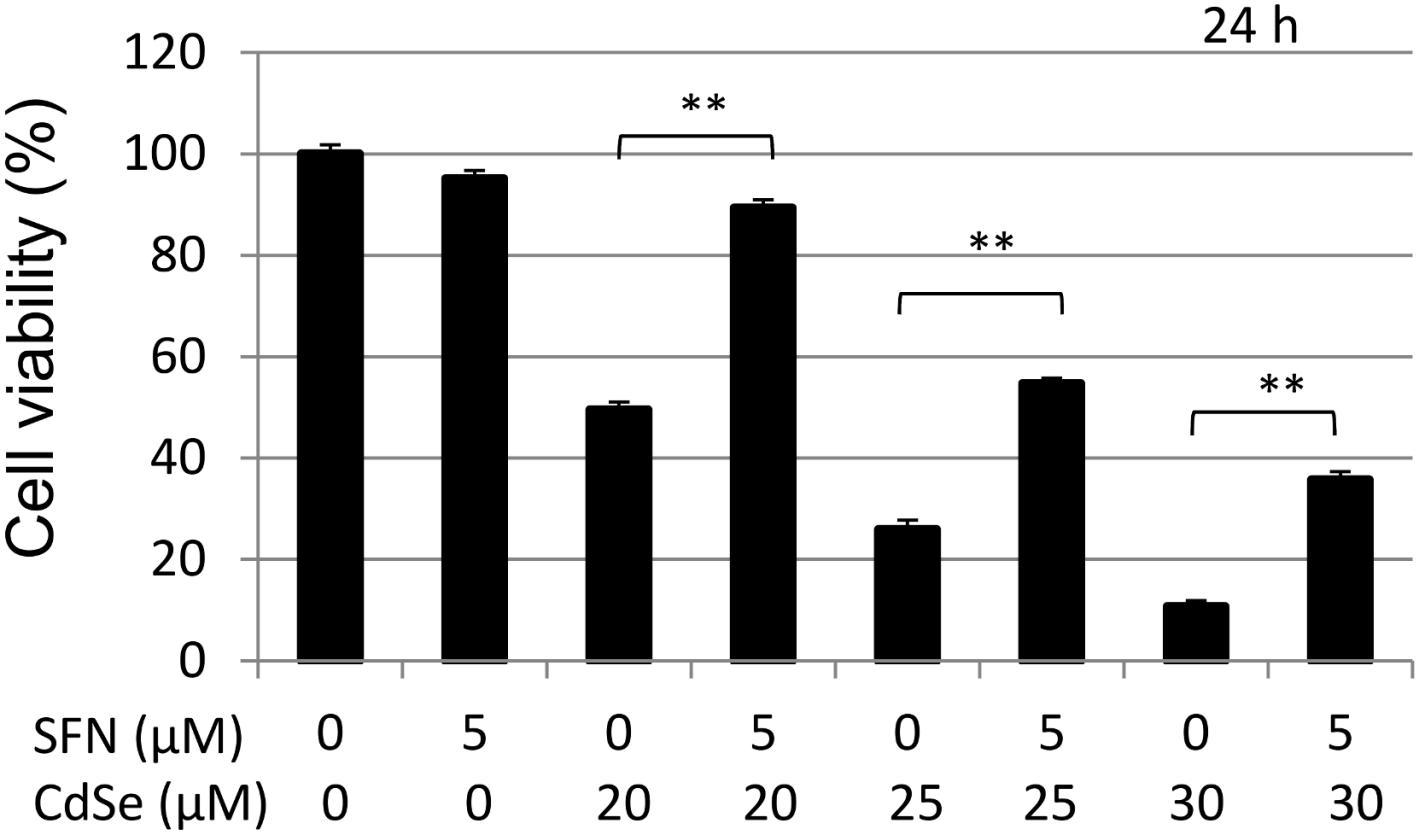
Effect of SFN on the cytotoxicity of CdSe QDs in HHL-5 cells. Effect of SFN pre-treatment on CdSe QD-induced cytotoxicity. HHL-5 cells were pre-treated with SFN (5 μM) for 24 h, and then incubated with 20–30 μM CdSe QDs for another 24 h. Cytotoxicity was measured by MTT assay, and data shown as means ± SD (n = 6). **P<0.01.

**Fig 2:**
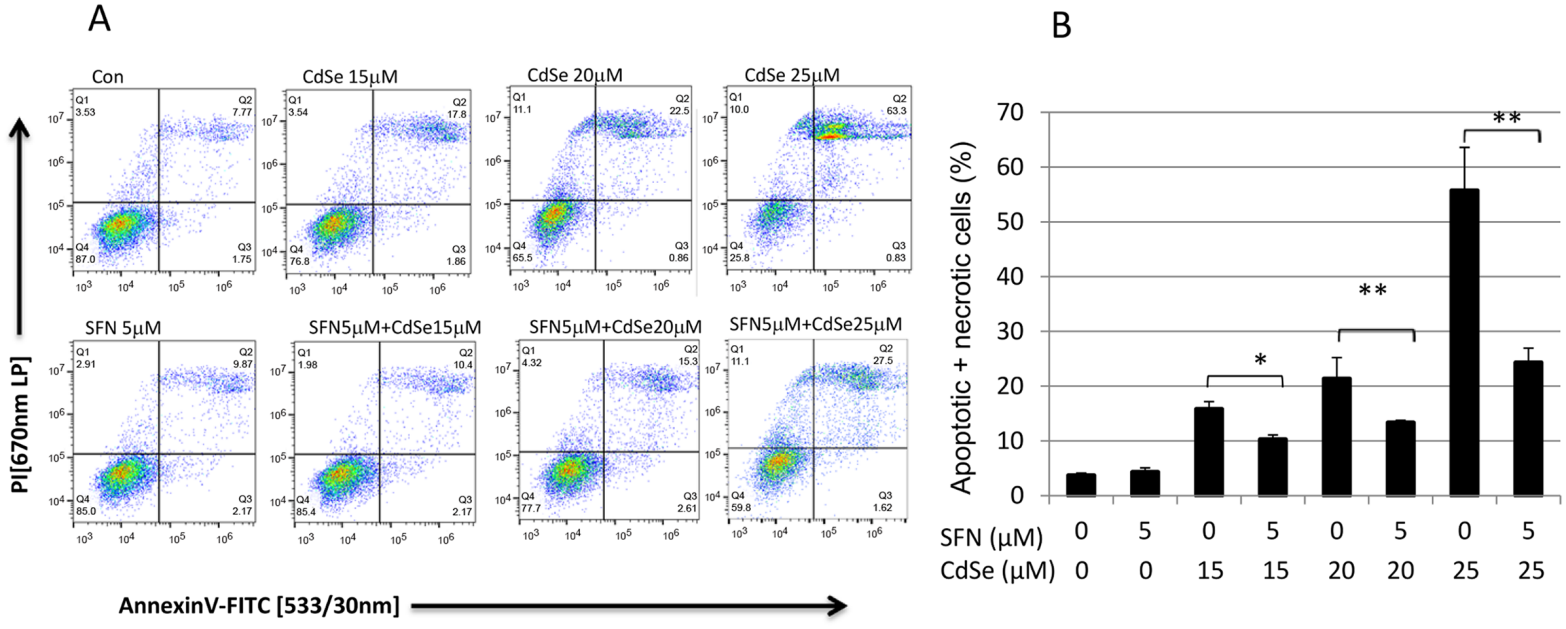
Cell death detection in CdSe-exposed and SFN pre-treated hepatocytes. (A) Pre-treatment with 5 μM SFN abrogated the protective effect with an observable increase the viable cell percentage (double negative, Q1) relative to the non-pre-treated cells. Samples were run on a BD Accuri C6 flow cytometer (Ex 488nm; Em 533/30nm BP & 670nm LP). Data is representative of at least 3 experiments. (B) Effect of SFN on CdSe QD-induced apoptosis and necrosis. Apoptosis and necrosis were determined by flow cytometry using Annexin V/PI staining. *P<0.05, **P<0.01.

CdSe (10:1) QDs showed notable cytotoxicity in HHL-5 cells after 12 h exposure. The cytotoxicity was more significant after 24 h with an IC_50_ = 20.4 μM CdSe pairs which is equivalent to 0.78 nmol core QDs/ml. However, when the cells were pre-treated with 5 μM SFN for 24 h, the cytotoxicity induced by 20 μM CdSe QDs (24 h exposure) significantly decreased, raising cell viability from 49.5 to 89.3% (P<0.01, Fig 1). Moreover, CdSe QDs (15–25 μM) caused a concomitant rise in the percentage of necrotic (PI positive cells) and putative late stage apoptotic cells (double positive), as indicated by Annexin V/PI staining (Fig 2). CdSe QD-associated-fluorescence could account for the majority of double positives observed. True Annexin V positive cells were observed at higher fluorescence levels in all samples. CdSe QD-associated fluorescence was limited in the PI channel and does not account for the two log decade shift in fluorescence seen in CdSe treated samples. Furthermore, absolute sample cell counts and the appearance of cellular debris indicated loss of cellular integrity in CdSe QD-treated samples. Thus, overall, the data indicated that CdSe QD exposure led to necrotic cell death. Pretreatment with 5 μM SFN abrogated cytotoxicity induced by CdSe QDs with an observable increase in the viable cell percentage (double negative) relative to the non-pretreated control cells (Fig 2) as well as increased absolute cell counts and minimal cellular debris.

### Effects of SFN on the intracellular levels of GSH

**Fig 3:**
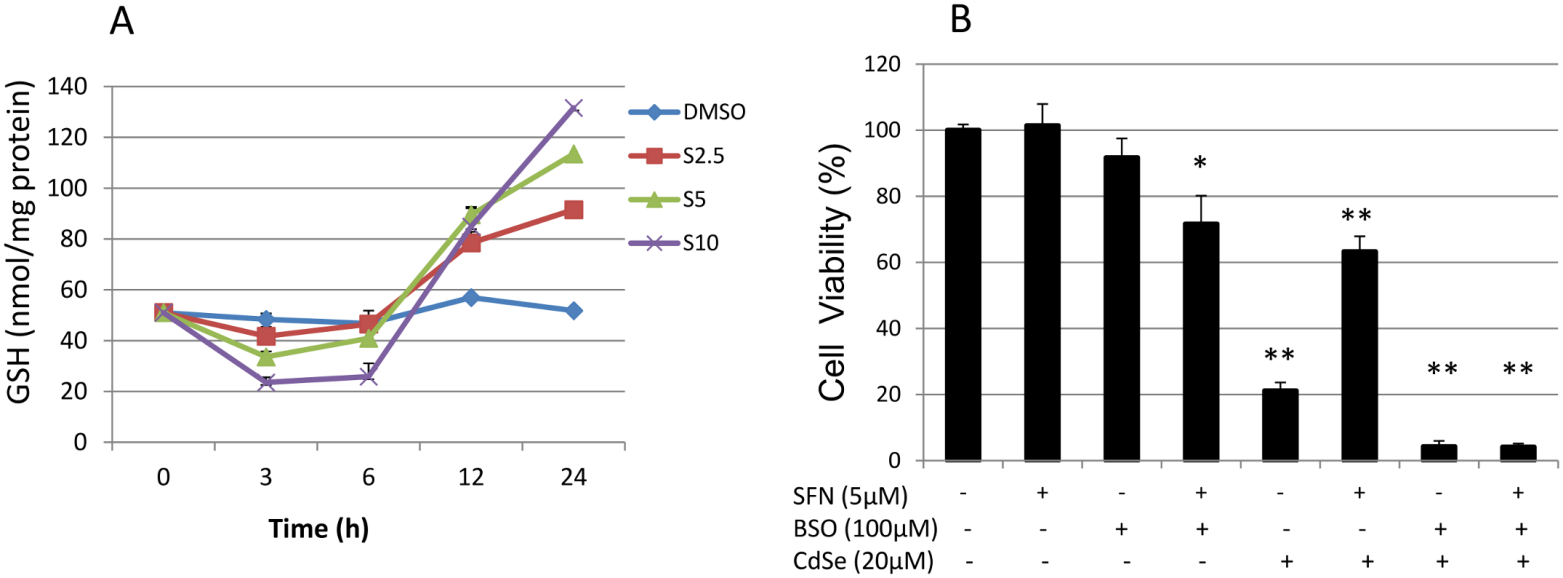
Intracellular GSH levels and susceptibility to CdSe QD-induced cytotoxicity. (A) Effect of SFN on intracellular GSH levels. HHL-5 cells were exposed to SFN (2.5, 5 and 10 μM) with DMSO (0.1%) as a control for 0, 3, 6, 12 and 24 h. The levels of intracellular GSH were measured by an HPLC assay. Data are shown as means ± SEM (n = 3). (B) Effect of SFN and BSO pre-treatment on CdSe QD-induced cytotoxicity. HHL-5 cells were pre-treated with SFN (5 μM) and/or BSO (100 μM) for 24 h, and then incubated with 20 μM CdSe QDs for another 24 h. Cytotoxicity was measured by MTT assay, and data shown as means ± SD (n = 6). *P<0.05, **P<0.01.

GSH is the most important and abundant endogenous antioxidant in mammals and its regulation represents an important research topic in chemoprevention [29]. Synthesis of the rate-limiting enzyme for glutathione synthesis, glutamate cysteine ligase (GCL), is regulated partly by the Keap1-Nrf2-antioxidant response element (ARE) pathway [30]. GSH has been shown to protect against cadmium-induced toxicity in cultured Chinese hamster cells [31]. In this study, SFN caused a dose dependent biphasic depletion and repletion of intracellular reduced GSH. SFN induced a pro-oxidant effect characterized by depletion of intracellular glutathione during short term exposure (3–6 h), followed by an antioxidant effect with up-regulation of glutathione at 24 h. The concentration of reduced GSH in control HHL-5 cells at time 0 was 51.0 nmol/mg protein. When cells were treated with 5 μM SFN for up to 24 h, the GSH level decreased to 33.6 nmol/mg protein at 3 h, 40.9 nmol/mg protein at 6 h, then the GSH levels increased to 89.5 and 113.5 nmol/mg protein at 12 and 24 h respectively, which were 1.7-, 2.2- fold of the control (Fig 3A). Moreover, at 10 μM SFN treatment the GSH levels decreased to 23.5 nmol/mg protein (46% of the control) at 3 h, 25.8 nmol/mg protein (50.6% of the control) at 6 h, whereas at 24 h the GSH level increased to 131.6 nmol/mg protein (2.6-fold of the control). However, when cells were pre-treated with L-buthionine S,R-sulfoximine (BSO) (100 μM, 24 h), a specific inhibitor of GCL, the toxicity of CdSe QDs was enhanced (cell viability from 63% decreased to 4.2%), and the protective effect of SFN pre-treatment on CdSe QD toxicity was completely abolished (Fig 3B). These data suggest that GSH exerts an important protective role in CdSe QD-mediated cell death.

### Effects of SFN on Nrf2 translocation and the expression of TR-1 and QR-1

The depletion of intracellular GSH is essential for SFN to facilitate the modification of Cys residues in Keap1. This enables Nrf2 to escape Keap1-dependent ubiquitination and degradation, and results in activation of Nrf2 [32, 33]. Incubation with increasing concentrations of SFN (2.5, 5 and 10 μM for 24 h) induced significant translocation of Nrf2 to the nucleus (4.7-, 9.7- and 18.2-fold over control cells respectively, S2A Fig). It is interesting that CdSe QDs alone also induced Nrf2 translocation into nucleus, and SFN-induced Nrf2 translocation can be enhanced by further treatment with CdSe QDs (S2B Fig). Nrf2, a master transcriptional factor of the endogenous anti-oxidant system, exerts chemoprotective effects via the induction of over 100 genes [34] including thioredoxin reductase (TR-1) and quinone reductase (QR-1). TR-1 is an important antioxidant enzyme catalysing the reduction of thioredoxin and H_2_O_2_. QR-1 is an important phase-II enzyme involved in detoxification of xenobiotics. HHL-5 cells were treated with 2.5, 5 and 10 μM SFN for 24 and 48 h (S3 Fig). SFN induced the expression of TR-1 and QR-1 in both a time- and dose-dependent manner. Treatment with SFN for 24 h increased the expression of TR-1 and QR-1 (1.8- and 4.5-fold respectively). These results are consistent with previous data showing up-regulation of TR-1 by SFN in HHL-5 cells measured by radioimmunoassay [28], and also with results obtained using Caco-2 and HepG2 cells [35, 36].

### Effect of knockdown TR-1, QR-1, Keap1 or Nrf2 on cytotoxicity of CdSe QDs and protective role of SFN

**Fig 4:**
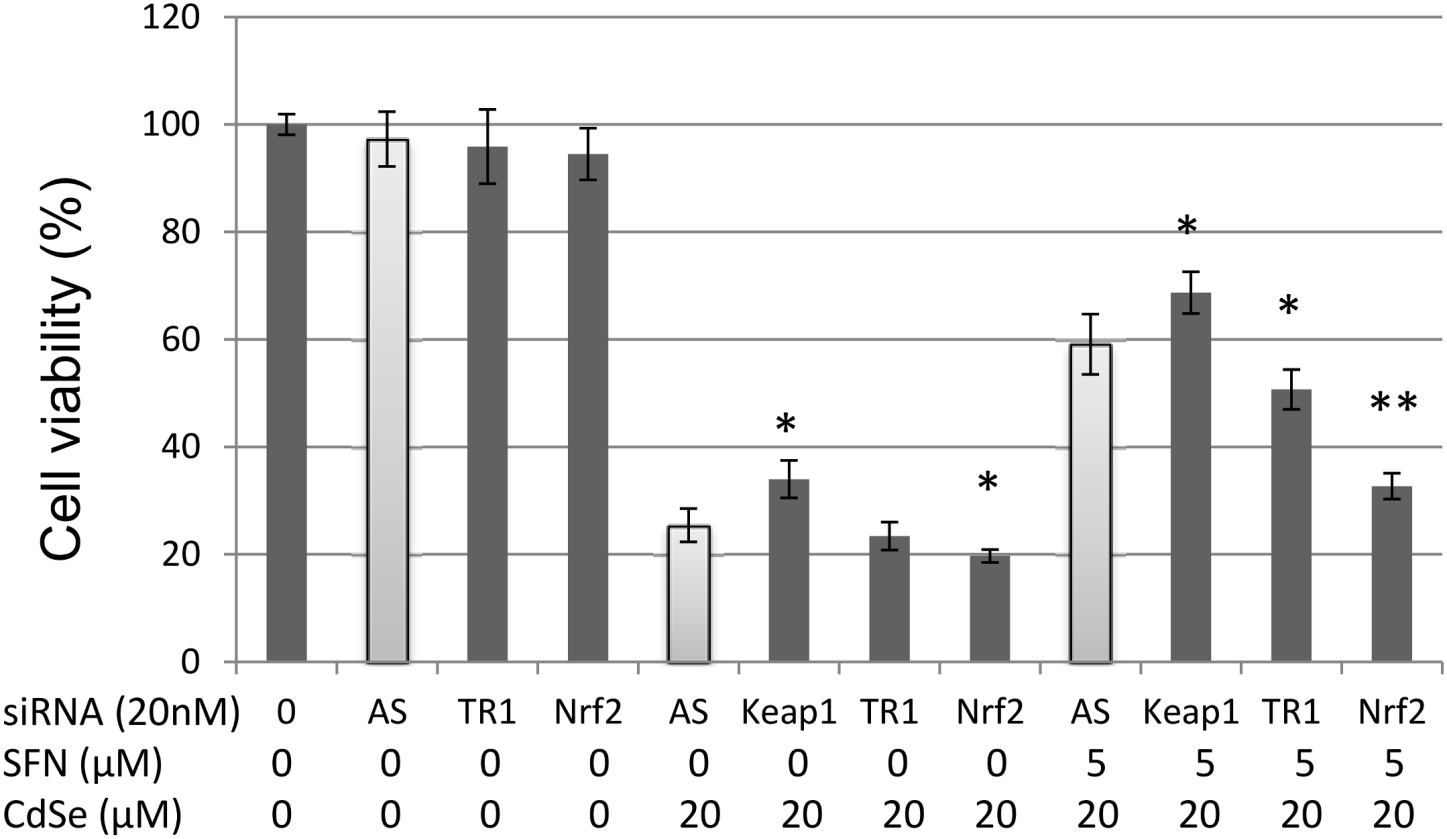
Effect of knockdown TR-1, Keap1 and Nrf2 on cytotoxicity in HHL-5 cells exposed to CdSe QDs. TR-1, Keap1 or Nrf2 were knocked down, respectively. Allstars (AS) were used as a negative control. Cells were incubated with 5 μM SFN or DMSO (0.1%) control for 24 h then exposed to 20 μM CdSe QDs for 24 h. Cell viability was measured by MTT assay, and data shown as means ± SD (n = 6). Significant levels (*P<0.05; **P<0.01) in comparison with AllStars (AS) negative controls.

TR-1 is driven by the Keap1-Nrf2-ARE signalling pathway. CdSe QDs (20 μM) decreased HHL-5 cell viability to 25.4% without SFN pretreatment. siTR-1 was found to have no significant effect on cell viability. However, siNrf2 knockdown indicated that diminished Nrf2 signalling enhanced the cytotoxicity of CdSe QDs, i.e. cell viability decreased from 25.4 to 19.7% (P<0.05) (Fig 4). In contrast, siKeap1, which enhances the Nrf2 pathway (S2 Fig) increased the cell viability to 34%. Pre-treatment with SFN (5 μM, 24 h), increased the cell viability to 59.1%. siTR-1 decreased CdSe QD-induced cell death from 59.1 to 50.7% (P<0.05, Fig 4); whereas siQR-1 has no effect on CdSe QD-induced cell death in HHL-5 cells (data not shown). Moreover, when Nrf2 was knocked-down, viable cell numbers decreased to 32.7% (P<0.01) indicating a significant abrogation of the protection provided by SFN against CdSe QD-induced cell death. In contrast, siKeap1 resulted in more Nrf2 translocation into nucleus, and siKeap1 plus SFN resulted in an enhanced protection (cell viability was increased to 68.7%, Fig 4). Taken together, these results demonstrate that Keap1-Nrf2-ARE signalling pathway plays an important role in CdSe QD-induced cell death.

### Effect of SFN and CdSe QDs on metallothionein mRNA transcription

Metallothionein (MT) is a small-molecular weight, cysteine-rich protein that binds metals, and it is known that MT is a Nrf2–driven gene since there is at least one ARE in the MT regulatory region [37]. The protective role of MT in cadmium toxicity has been well established [31, 38], and SFN is a known inducer of MT [39]. In this study, SFN (5 μM) induced MT-1A mRNA by 2.49-fold; CdSe QDs (20 μM) induced 17.12-fold, pre-treatment with SFN (5 μM) then exposure to CdSe QDs (20 μM) induced MT-1A synergistically (up to 47.15-fold, S4 Fig). CdSe QD-induced MT-1A mRNA transcription may be due to a potential release of Cd^2+^ ions from the CdSe core and the physicochemical characteristics of the CdSe QDs themselves.

### Effect of SFN on activation of autophagy

**Fig 5:**
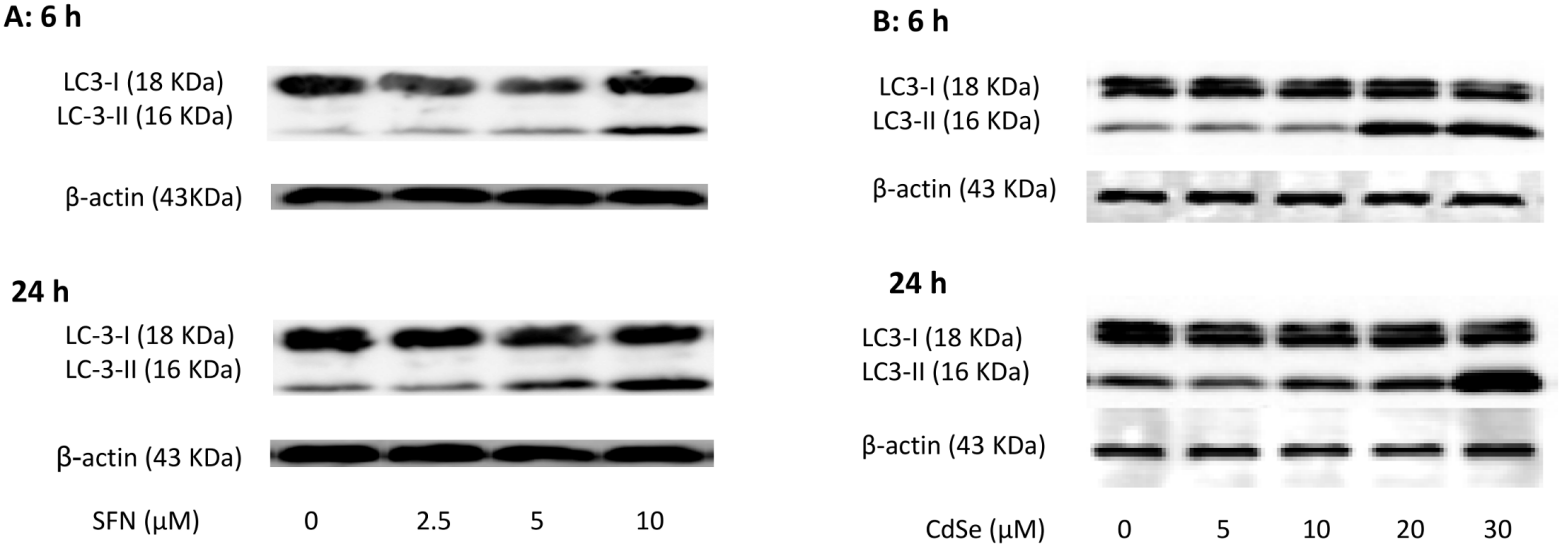
Induction of LC3-II by SFN and CdSe QD in HHL-5 cells. HHL-5 cells 48 h after seeding were (A) exposed to SFN for 6 or 24 h, or (B) treated with varying levels of CdSe QD for 6 or 24 h. The expression of LC-3-II was analysed by Western blot analysis.

Deceased cellular GSH levels activate autophagy [40], and SFN is a known inducer of autophagy in cultured tumour cells [25]. Autophagy involves the formation of autophagosomes, which encapsulate cytoplasm and organelles and fuse with lysosomes, leading to the degradation of the contents of the autophagosome [41]. Light chain protein 3-II (LC3-II) is the major protein of the autophagosome membrane. LC3 has two forms: LC3-I is cytosolic, whereas LC3-II is membrane-bound. During autophagy, LC3-I is converted to LC3-II and increased levels of LC-3II correlate with the extent of autophagosome formation [42]. SFN induces autophagy in different cells, such as human breast cancer cells [43] and human colon cancer cells [44]. In this study, SFN induced LC3-II production in HHL-5 cells in a dose- and time-dependent manner (Fig 5A). Western blot analysis showed that SFN at 5 and 10 μM increased LC3-II (16 kDa) production 2- to 3-fold (6 h), and at 24 h this increased to 3- and 7-fold, respectively, compared to corresponding controls. When cells were incubated with CdSe for 6 or 24 h, LC3-II was induced by 20–30 μM CdSe (Fig 5B). The results suggest CdSe QDs activate autophagy in human hepatocytes. The interplay between SFN-induced autophagy and apoptosis has been reported in cultured tumour cells, and the inhibition of autophagy can enhance SFN-induced tumour cell death [25, 43, 44].

### Effect of wortmannin or 3-MA on the effect of SFN on CdSe QD-induced cell death

**Fig 6:**
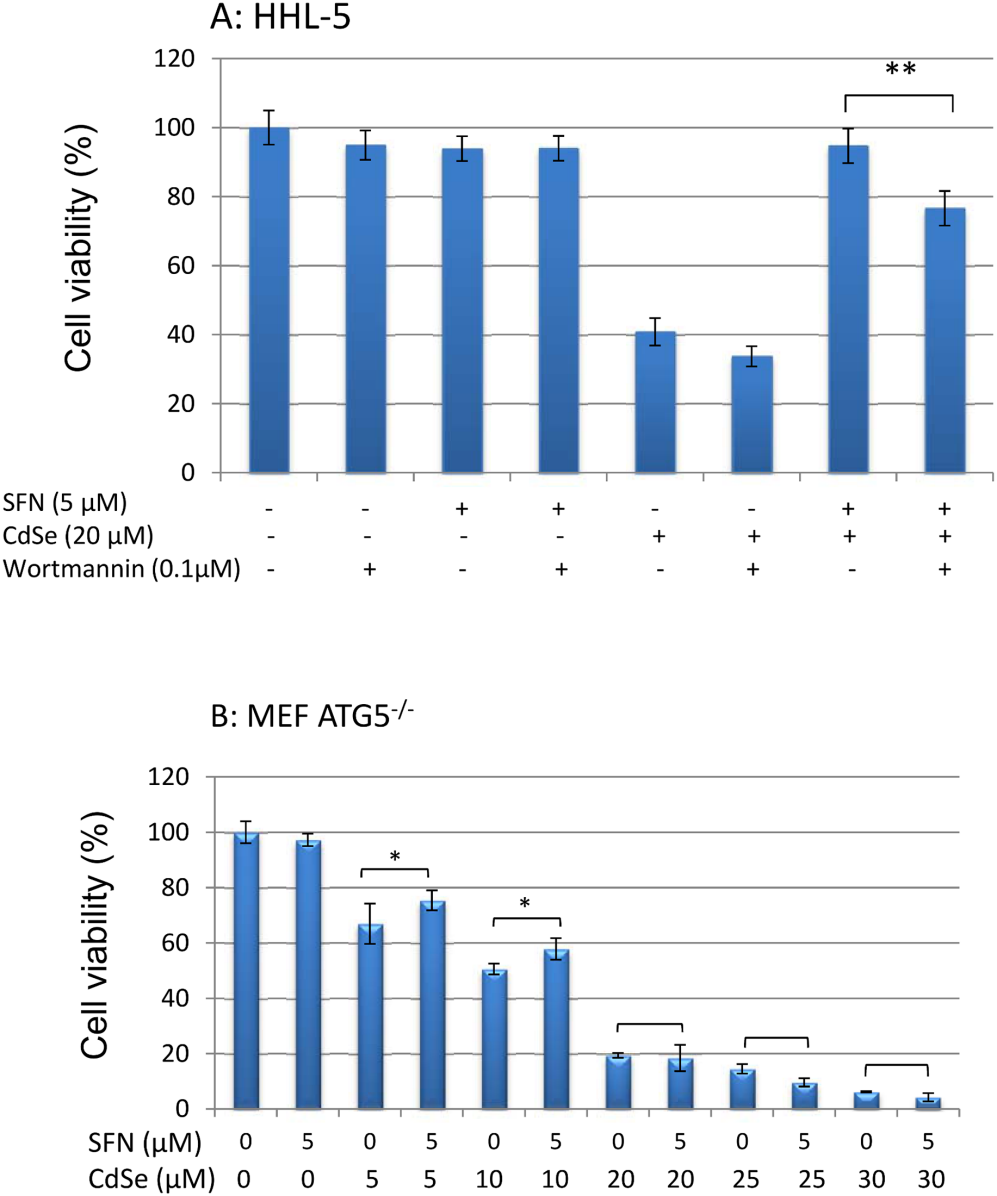
Effect of autophagy inhibitor on the protective effect of SFN on CdSe QD-induced cell death. (A) HHL-5 cells were pre-incubated with wortmannin (0.1 μM) for 6 h, and then exposed to SFN (5 μM) for 24 h. There was a further 24 h exposure with 20 μM CdSe QDs. (B) MEF ATG5^-/-^ cells were pre-incubated with SFN (5 μM) for 24 h and then exposed for a further 24 h with CdSe QDs (10–30 μM). Cytotoxicity was measured by MTT assay. Data are shown as means ± SD (n = 6) (**P<0.01).

Wortmannin acts as a selective inhibitor of type III PI-3K [45]. When wortmannin (0.1 μM) was used to inhibit SFN-induced autophagy, the protective effect of SFN (5 μM) on CdSe QD (20 μM)-induced cell death was suppressed significantly and cell viability decreased from 95 to 78% (Fig 6A). A similar effect was observed using 3-MA, another commonly used autophagy inhibitor, which can also block autophagosome formation via inhibition of type III PI-3K [46]. 3-MA decreased the protective effect of SFN on cell viability from 86.3% to 68.4% (S5 Fig). These results suggest that the induction of autophagy by SFN has a significant role in the protection against CdSe-induced cell death. The role of autophagy was further examined using ATG5^-/-^ MEF that lack autophagy-associated gene 5 (ATG5) which is essential for autophagy. Pre-treatment of ATG5^-/-^ MEF with SFN (5 μM for 24 h) did not protect against cell death induced by CdSe QDs (20–30 μM) signifying the importance of autophagy in the protection (Fig 6B); whereas at low levels of CdSe QDs (5–10 μM) exposure there was a protective effect (7.2–7.5% increase in cell viability, P<0.05) which may be due to the activation of Nrf2 pathway.

### SFN protected against the CdSe QD-induced acute liver damage

**Fig 7:**
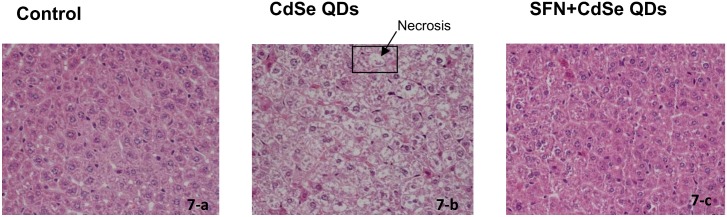
The protective effect of SFN against the hepatoxicity of CdSe QDs in mouse liver. Liver toxicity in mice was evaluated by histological examination after H&E staining. CdSe QDs caused hepatocellular necrosis with cell swelling and modulated nuclear morphology as indicated by the arrow. The original magnification was 400X.

Liver toxicity in mice was evaluated by histological examination after H&E staining (Fig 7). In the control group, the hepatocytes were arranged in regular rows of hepatic cords; there was no hepatic sinusoid congestion nor were there any abnormal changes in liver cells (Fig 7-a). However, in the group exposed to CdSe QDs, hepatocellular ballooning degeneration occurred over large areas (Fig 7-b). Many hepatocytes have diffusion in cytoplasm and look less nucleated possibly because of nuclear condensation, break up and loss (pyknosis, karyorrhexis and karyolysis). If this state continues to develop, it would lead to hepatocellular necrosis. Interestingly and as predicted, the SFN-protected group incurred almost no cell death and the liver histology was similar to that of control groups (Fig 7-c).

## Discussion

SFN is an isothiocyanate derived from cruciferous vegetables such as broccoli and cauliflower and its anti-cancer activity was discovered 20 years ago [47]. It has been extensively studied since it is an activator of Nrf2 [34, 48–50]. Human intervention studies suggest that the levels of ITCs and their metabolites could reach approximately 2 μM in plasma following ingestion of 200 μmol of ITCs [51]. The highest level reported was 7.3 μM after consumption of 100 g high-glucosinolate broccoli containing 345 μmol SFN and SFN metabolites [52]. SFN chemoprevention is currently a popular subject for study. There are at least 20 registered human trials listed at www.clinicaltrials.gov that are examining the effectiveness of SFN or broccoli sprout preparations (a source of SFN) in the treatment of various diseases including cancer, virus infection and chronic obstructive pulmonary disease. The cytotoxicity of CdSe QDs presents a barrier to their clinical applications. There are some less potentially toxic QDs available such as PhosphorDots (http://www.nanomaterialstore.com/nano-phosphor.php). In the present study, we have shown that SFN at a physiologically relevant concentration (5 μM) protects against CdSe QD-induced cytotoxicity in immortalized human hepatocytes. The mechanisms include SFN modulation of cellular GSH levels since BSO treatment abolished the protective effect of SFN (Fig 3B).

Although the two antioxidant enzymes, TR-1 and QR-1, were shown to be less significant in the protection against CdSe QDs toxicity, the role of Nrf2 in protection was prominent. There are many other Nrf2-ARE driven antioxidant enzymes such as haem oxygenase-1 (HO-1), glutathione peroxidases (GPXs), GSTs, and peroxiredoxin, which may also be involved in the protection against oxidative stress [53, 54]. SFN is known to up-regulate p62, a protein that binds ubiqutinated proteins and delivers them to autophagosomes for degradation. More interestingly, both SFN and CdSe QDs induced MT-1A mRNA transcription and there is a synergistic effect between SFN and CdSe DQs. The observation that CdSe QD-induced MT-1A mRNA expression suggests that there is a potential release of cadmium ions from the core [7]. Although the up-regulation of MT-1A may be due to the physicochemical characteristics of the CdSe QDs per se, it would be of interest to see if MT expression could be induced by other types of QDs, such as non-CdSe and capped CdSe QDs. So far, there is only one study that has correlated CdSe QD exposure with the induction of HO-1 [55]. The mechanism underlying CdSe QD-mediated biological influences might derive from free cadmium ions liberated from QDs, from the toxicity of QD particles themselves or a combination of both [6]. There are reports that SFN exerts selective cytotoxicity towards cancer cells via the production of ROS, however, an increase in oxidative stress appears to trigger an adaptive response [56, 57]. From the current study, there is also a Nrf2-independent mechanism whereby SFN activates autophagy and enhances the protective effect of SFN on CdSe QD-induced cell death. The protective role of autophagy has been confirmed using autophagy inhibitors such as wortmannin, 3-MA, and in ATG5^-/-^ MEF (Fig 6). Moreover, in the animal experiment, CdSe QDs caused significant liver damage in mice, and administration of SFN significantly decreased the liver toxicity caused by CdSe QD exposure. Furthermore, SFN has also been shown to protect against hepatotoxicity induced by toxins and drugs such as microcystin, cisplatin, triptolide and aflatoxin B1, and most of the protection were attributed to the induction of Nrf2 and phase II enzymes [58–61].

In summary, SFN treatments provided protective effects on CdSe QD-induced cytotoxicity in human hepatocytes and the livers of the mice. The mechanisms of protection were mainly via activation of Nrf2-ARE and autophagy pathways that potentiate the protective effect of SFN against CdSe QD-induced cell death in hepatocytes.

## Materials and Methods

### Reagents

SFN, 4-methylsulfinylbutyl isothiocyanate was purchased from Alexis Biochemicals (UK). Methylthiazolyldiphenyl-tetrazolium bromide (MTT), wortmannin, 3-methyladenin (3-MA), cadmium perchlorate, dimethyl selenourea, LC3 antibody and GenElute™ total mammalian RNA isolation kit were all purchased from Sigma-Aldrich (UK). Complete protease inhibitors were obtained from Roche Applied Science (UK). Rabbit polyclonal primary antibodies to Nrf2, TR-1, QR-1 goat polyclonal primary antibody to Keap1, β-actin, rabbit polyclonal primary antibody to Sam68 (Src-associated in mitosis 68 kDa, a RNA binding protein), HRP-conjugated goat anti-rabbit and rabbit anti-goat IgG were all purchased from Santa Cruz Biotechnology (Heidelberg, Germany). siRNAs for Nrf2, TR-1, QR-1, Keap1 and AllStars negative control were all purchased from Qiagen (West Sussex, UK). Annexin V-FITC apoptosis detection kit was purchased from eBioscience (UK). Electrophoresis and Western blotting supplies were obtained from Bio-Rad (UK), and the Chemiluminescence kit was from GE Healthcare (Little Chalfont, UK).

### Synthesis of CdSe NPs

CdSe nanoparticles were synthesized by the microwave heating of an aqueous solution of cadmium perchlorate (CdCl_2_O_8_) as a source of cadmium ions with N,N-dimethyl selenourea (C_3_H_8_N_2_Se) as a source of selenium ions, in the presence of sodium citrate as a stabilizer [1, 62]. In brief, 50 mg of sodium citrate was dissolved in 45 ml of Milli-Q water, and then the pH was adjusted to 9.2. 2 ml of 0.01 M CdCl_2_O_8_ and 2 ml of 0.01 M C_3_H_8_N_2_Se were then added and the pH adjusted to 9.2 again. The mixture of precursors was heated in a conventional microwave oven at 800W continuously for 75s and then stored in the dark at room temperature for 3 days. Adding 2 ml 0.01 or 0.1 M CdCl_2_O_8_ resulted in CdSe QDs (1:1) and (10:1) respectively. The diameters of the CdSe QD particles were 6.7±1.7 nm as measured by transmission electron microscopy (TEM) imaging (S1 Fig). The total concentration of CdSe pairs in each preparation (400 μM) was determined assuming that all of the Se^2-^ in the C_3_H_8_N_2_Se reacted to form CdSe pairs. CdSe (10:1) QDs have 26 CdSe pairs per particle, and the initial concentration of CdSe core particles was 15.38 μM QDs, i.e. 1 nmol CdSe core QDs/ml = 26 μM CdSe pairs [1]. In the following experiments, the concentration of CdSe pairs, 5–20 μM, was used.

### Cell culture

Immortalised human hepatocytes (defined as HHL-5) were kindly supplied by Dr A. Patel, Medical Research Council (MRC) Virology Unit (Glasgow, UK). The primary hepatocytes were immortalised with Moloney’s mouse leukaemia virus and contained markers of hepatocyte and biliary phenotype, cytochrome P450 and albumin [63]. HHL-5 cells were cultured in DMEM supplemented with foetal bovine serum (10%), 2 mM glutamine, penicillin (100 U/ml) and streptomycin (100 μg/ml) under 5% CO_2_ in air at 37°C. When the cells achieved 70–80% confluence, they were exposed to various concentrations of CdSe and SFN (dissolved in DMSO) for different times, with DMSO (0.05%) as control. When HHL-5 cells were treated with the autophagy inhibitors, wortmannin or 3-MA, they were added 6 h prior to treatment with SFN. Mouse embryonic fibroblasts (MEF) lacking Atg5 gene expression (ATG5^-/-^) were a kind gift from Dr Noboru Mizushima at Tokyo Medical and Dental University, Japan [64]. MEF were cultured in the same medium as HHL-5.

### Cell viability assay

The MTT cell proliferation assay was used to detect the toxicity of CdSe QDs and the protective effect of SFN. HHL-5 cells were seeded in 96-well plates in DMEM with 10% FCS at a concentration of 0.5–1.0 × 10^4^ cells in a final volume of 100 μl per well. SFN treatments and controls had an equal final concentration of DMSO (0.05%). After 24 h treatment with SFN, cells were exposed to different concentrations of CdSe QDs in fresh medium for a further 24 h period. After treatment, the medium was removed, 100 μl (5 mg/ml) MTT was added, and incubated at 37°C for 1 h to allow the MTT to metabolize. The formazan formed was then re-suspended in 100 μl DMSO per well. The final absorbance was recorded using a microplate reader (BMG Labtech Ltd, UK) at a wavelength of 550 nm and a reference wavelength of 650 nm.

### Knockdown TR-1, QR-1, Keap1 and Nrf2 by siRNA

HHL-5 cells were seeded on 96-well plates at density of 1×10^4^/well in DMEM with 10% FCS. After 24 h, the cells were transfected with siRNA for TR-1, QR-1, Keap1, Nrf2 or Allstars (no homology to any known mammalian gene). Briefly, the cell culture medium was replaced with 100 μl medium containing12% FCS, then 10 nM siRNA with 0.6 μl Hiperfect transfection reagent (in 20 μl medium without serum and antibiotics) was incubated at room temperature for 10 min before being gently added drop-wise to the cells. AllStars was used as a negative control siRNA. After 24 h incubation with siRNAs, protective compound SFN was added in fresh medium for a further 24 h, then the effect of CdSe QDs (20 μM, 24 h) on cell death was measured using the MTT cell viability assay. The siRNA knockdown efficiency of Nrf2 and Keap1 was characterized using Western blot analysis (S2C and S2D Fig). siRNA knockdown of TR-1 in HHL-5 cells has been described in a previous publication [28].

### Protein extraction and Western blot analysis

For total protein, HHL-5 cells were washed twice with ice-cold PBS, harvested by scraping in 20 mM Tris-HCl (pH 8), 150 mM NaCl, 2 mM EDTA, 10% glycerol, 1% Nonidet P40 (NP-40) containing mini-complete proteinase inhibitor and 1 mM PMSF and then incubated in an ice bath for 20 min and centrifuged at 12,000 g for 15 min at 4°C. Supernatant was collected and the protein concentration determined by the Brilliant Blue G dye-binding assay of Bradford using BSA as a standard. For the nuclear protein, the extraction was performed using a Nuclear Extract Kit (Active Motif, UK), following the manufacturer’s instructions. Protein extracts were heated at 95°C for 5 min in loading buffer and loaded onto 10% SDS-polyacrylamide gels together with a molecular weight marker. After routine electrophoresis and transfer, the PVDF membrane was blocked with 5% fat free milk in PBST (0.05% Tween 20) for 1 h and incubated with a specific primary antibody in 5% milk in PBST for 1 h. The membrane was washed three times for 45 min with PBST and then incubated with the secondary antibody diluted with 5% milk in PBST for 1 h. After further washing the membrane three times for 45 min with PBST, antibody binding was determined by a Chemiluminescence detection kit and densitometry was measured by Fluor Chem Imager (Alpha Innotech, San Leandro, CA).

### HPLC analysis of intracellular GSH

Intracellular concentrations of reduced GSH were determined by HPLC analysis of cell lysates derivatised with monobromobimane (mBBr). The procedure was adapted from Newton and Fahey [65], and Quievryn and Zhitkovich [66]. Approximately 1×10^6^ human hepatocytes were collected from 6-well plates, washed twice in PBS and suspended in 75 μl PBS containing 5 mM diethylenetriaminepentaacetic acid. The suspensions were acidified by addition of 300 μl 50 mM methanesulfonic acid, and then subjected to three freeze-thaw cycles alternating between liquid N_2_ and a 37°C heat block. GSH-containing supernatants were obtained after centrifugation at 12,000 g for 10 mins. For GSH derivatization, reactions contained 75 μl cell extracts and 25 μl pre-mixed buffer (final reaction concentration 50 mM HEPES, pH 8.0, 5 mM EDTA, 15 mM NaOH and 2 mM mBBr). The reaction was immediately vortexed and incubated for 15min in the dark at room temperature. After acidification with 1 μl 5 M methanesulfonic acid, the samples were centrifuged at 12,000 g. The supernatants were diluted three-fold in 10 mM methanesulfonic acid and analyzed by HPLC. Bimane-derivatised GSH (GSmB) was separated by HPLC on a HiChrom ACE-AR C_18_ 4.6×250 mm (5 μm) column with Solvent A (0.25%, v/v acetic acid and 10% methanol, pH 4). Detection was carried out with a Jasco fluorescence detector with excitation at 385 nm and emission at 460 nm. The level of GSH was expressed as nmol/mg of cellular soluble protein. The protein concentrations of cell extracts were determined by Bradford assay using BSA as standard (Sigma, UK).

### Quantitative real-time PCR analysis of MT-1A

Total RNA was extracted from HHL-5 cells using GenElute™ total mammalian RNA kit (Sigma, UK) according to the manufacturer's instruction. First strand cDNA was synthesised with 1 μg of total RNA using qScript cDNA SuperMix (Quanta BioSciences, UK). MT-1A mRNA quantification was determined by TaqMan real-time PCR using the Roche LightCycler 480 System (Roche Diagnostics, Switzerland). Forward primer, 5′-CTCCTGCTGCCCCATGAG-3′; reverse primer, 5′-TCTCTGATGCCCCTTTGCA-3′; probe, 5′-CCAAGTGTGCCCAGGGCTGCA-3′. The probe was labelled with a 5′ reporter dye, FAM (6-carboxyfluoroscein) and 3′ quencher dye, TAMRA (6-carboxytetramethylrhodamine). Real-time PCR reactions were carried out using Precision™ MasterMix (Primer Design, UK) and samples were run following a 10 min hot start at 95°C, followed by 40 cycles of denaturing at 95°C for 15 s and annealing/extension at 60°C for 60 s. Data were normalised against an invariant endogenous control, 18S ribosomal RNA. Forward primer 5′-GGCTCATTAAATCAGTTATGGTTCCT-3′, reverse primer 5′-GTATTAGCTCTAGAATTACCACAGTTATCCA-3′, probe 5′-TGGTCGCTCGCTCCTCTCCCA-3′. The threshold cycle number (Ct) obtained was converted into fold of relative induction using the ΔΔCt method.

### Flow Cytometry for apoptosis/necrosis

HHL-5 cells were seeded on 12-well plates at a density of 5×10^4^ cells per well and incubated at 37°C for 48 h. After treatment with 5–10 μM SFN for 24 h, cells were exposed to 10–20 μM CdSe NPs for 6 and 24 h. Cells were then detached from the wells using trypsin and collected by centrifugation at 180 g for 6 min at 4°C, and the pellets washed with cold PBS before being re-suspended in 400 μl cold PBS. Flow cytometry was performed with a BD Accuri C6 Flow Cytometer using 488 nm excitation with 533/30 nm band pass (BP) and 670 nm long pass (LP) filtered detection. The effect of CdSe QD on apoptosis was assessed using an Annexin V-FITC apoptosis detection kit (eBioscience, UK), according to the manufacturer's instructions. Cells were trypsinised and collected, Annexin V-FITC (fluorescein isothiocyanate) was used to label the apoptotic cells and propidium iodide (PI) used to stain the necrotic cells. For each sample 10,000 events were collected and the data were analysed using FlowJo software (Treestar Inc. USA).

### Protective effect of SFN on CdSe QD-induced liver damage in mice

ICR mice were obtained from the experimental animal centre in Heilongjiang University of Chinese Medicine, and maintained in a Specific Pathogen Free animal house according to the guidelines of the Institutional Animal Care Use Committee. This study was approved by the Animal Experimental Ethics Committee, Heilongjiang University of Chinese Medicine, Harbin, China (License No: SCXK-Hei-2012016). Regarding the acute exposure to CdSe QDs, 36 mice (BW 20-23g) were randomized into control, CdSe, and SFN treatment + CdSe groups with 12 mice (6 male and 6 female) in each group. Mice in the CdSe group were administrated intraperitoneally with 0.2 ml 400 μM CdSe QDs once. During the first 4 hours after administration, mouse reactions such as twitch, secretions from the eyes and nose, respiration and heartbeat rate were monitored. Mice in the SFN protection group were given SFN (40 mg/kg BW) every other day for 14 days through oral gavage (three doses before CdSe injection and four after CdSe injection in every other day). Control mice were given PBS orally only. Mice were then sacrificed 24 h after the last gavage of SFN through peritoneal injection with 10% chloral hydrate (anaesthetic agent) solution (0.3 ml/100g BW). During the whole experiment, there were no animals died of liver damage. The liver tissues were fixed in 10% buffered formal saline for 48 h before processing according to the defined pathologic protocols [67].

### Statistics

Data are represented as the mean ± SD or SEM. The differences between the groups were examined using the one-way ANOVA test, or Student’s t-test. A *p* value <0.05 was considered statistically significant. The IC_50_ value of SFN was determined using CalcuSyn Software (Biosoft, UK).

## Supporting Information

S1 Fig Transmission electron microscopy (TEM) image of CdSe QDs. (A) A drop of QDs solution was cast onto a carbon film grid prior to the measurement using a JOEL 2000EX TEM with the accelerating voltage of 200 kV. (B) Histogram shows the size distribution of CdSe QDs obtained by measuring 100 QDs from different parts of the grid.
(TIF)

S2 Fig Effect of SFN and CdSe QDs on Nrf2 translocation into nucleus, and siRNA knockdown. (A) HHL-5 cells were treated with SFN for 24 h. (B) cells were pre-treated with SFN (5 μM) for 24 h then treated with CdSe for further 24 h. DMSO (0.1%) was used as a control. (C) siRNA knockdown Keap1 and Nrf2 in HHL-5 cells. Cells were seeded into 10cm dish. After 24 h, cells were treated with siKeap1 or siNrf2. Allstars (AS) was used as a negative control. After 24 h treatment, medium was changed and 5 μM SFN or DMSO (0.05%) was added for further 24 h. Nrf2 in nuclear extract and Keap1 in cytosol (D) were detected using Western blot analysis.
(TIF)

S3 Fig Induction of TR-1 and QR-1 by SFN in HHL-5 cells. After seeding for 48 h, cells were exposed to SFN for 24 h (A) or 48 h (B). The expression of TR-1 and QR-1 were analysed by Western blot analysis. The band density was quantified using the Quantity One^®^. Data are the average of 3 experiments (±SD).
(TIF)

S4 Fig Effect of SFN and CdSe QDs on MT-1A mRNA transcription. HHL-5 cells were treated with either SFN (5 μM), or CdSe (20 μM) and in their combination, i.e. pre-treatment SFN + CdSe for 24 h. DMSO (0.1%) as control. Total RNA was isolated using a GenElute™ total mammalian RNA kit (Sigma, UK). MT-1A mRNA was determined by TaqMan real-time PCR assays. The bar graphs represent means ± SD of three replicates. Statistical significance from the control, **p< 0.01.
(TIF)

S5 Fig Effect of 3-MA on the effect of SFN on CdSe QD-induced cell death. HHL-5 cells were pre-incubated with 3-MA (5 mM) for 6 h and then exposed to 5 μM SFN for 24 h. There was then a further 24 h exposure with 20 μM CdSe QDs. Cytotoxicity was measured by MTT assay. Data are shown as means ± SD (n = 6) (**P<0.01).
(TIF)
