# Supplementary figures and images for: Sulforaphane Protects the Liver against CdSe Quantum Dot-Induced Cytotoxicity
Source: PLoS One. 2015 Sep 24;10(9):e0138771. doi: 10.1371/journal.pone.0138771 (PMC4581733; doi:10.1371/journal.pone.0138771)

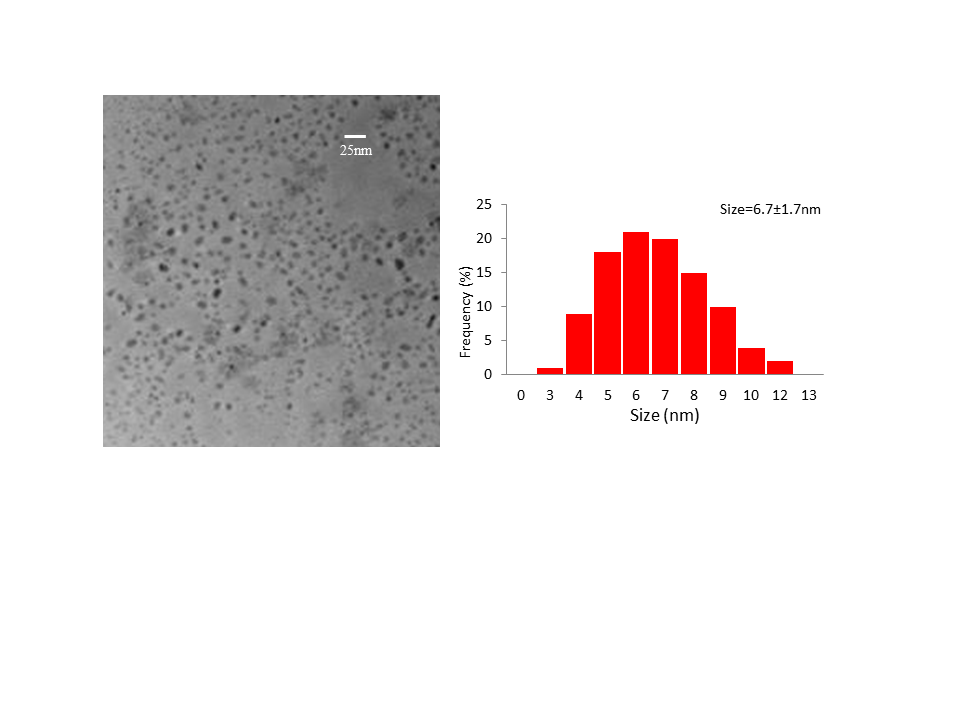

Supplement: S1 Fig — (A) A drop of QDs solution was cast onto a carbon film grid prior to the measurement using a JOEL 2000EX TEM with the accelerating voltage of 200 kV. (B) Histogram shows the size distribution of CdSe QDs obtained by measuring 100 QDs from different parts of the grid. (TIF) [file pone.0138771.s001.TIF]

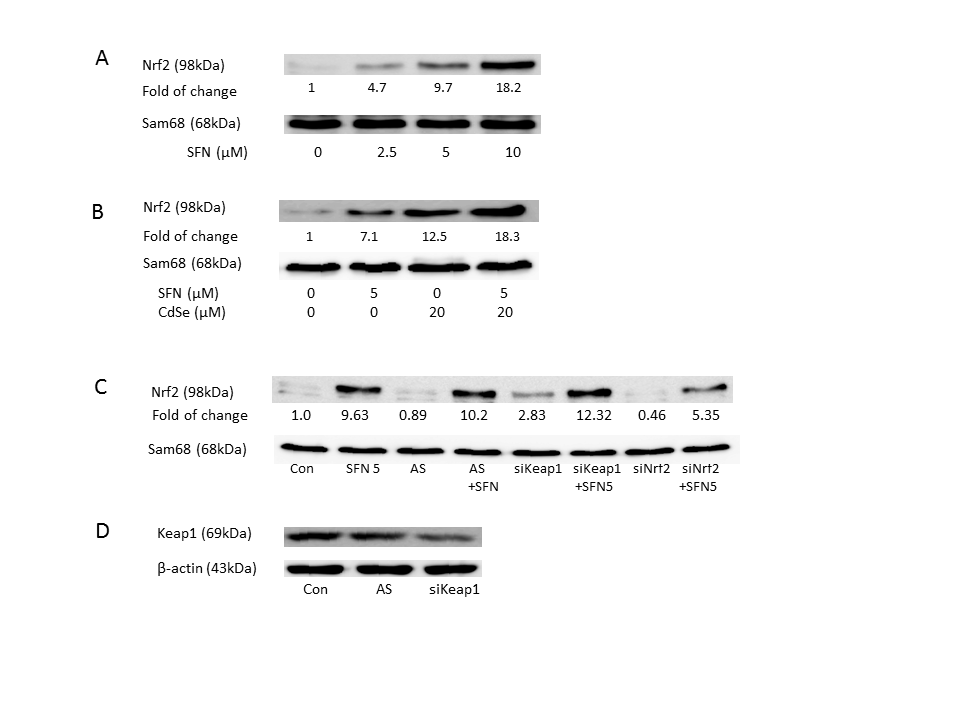

Supplement: S2 Fig — (A) HHL-5 cells were treated with SFN for 24 h. (B) cells were pre-treated with SFN (5 μM) for 24 h then treated with CdSe for further 24 h. DMSO (0.1%) was used as a control. (C) siRNA knockdown Keap1 and Nrf2 in HHL-5 cells. Cells were seeded into 10cm dish. After 24 h, cells were treated with siKeap1 or siNrf2. Allstars (AS) was used as a negative control. After 24 h treatment, medium was changed and 5 μM SFN or DMSO (0.05%) was added for further 24 h. Nrf2 in nuclear extract and Keap1 in cytosol (D) were detected using Western blot analysis. (TIF) [file pone.0138771.s002.TIF]

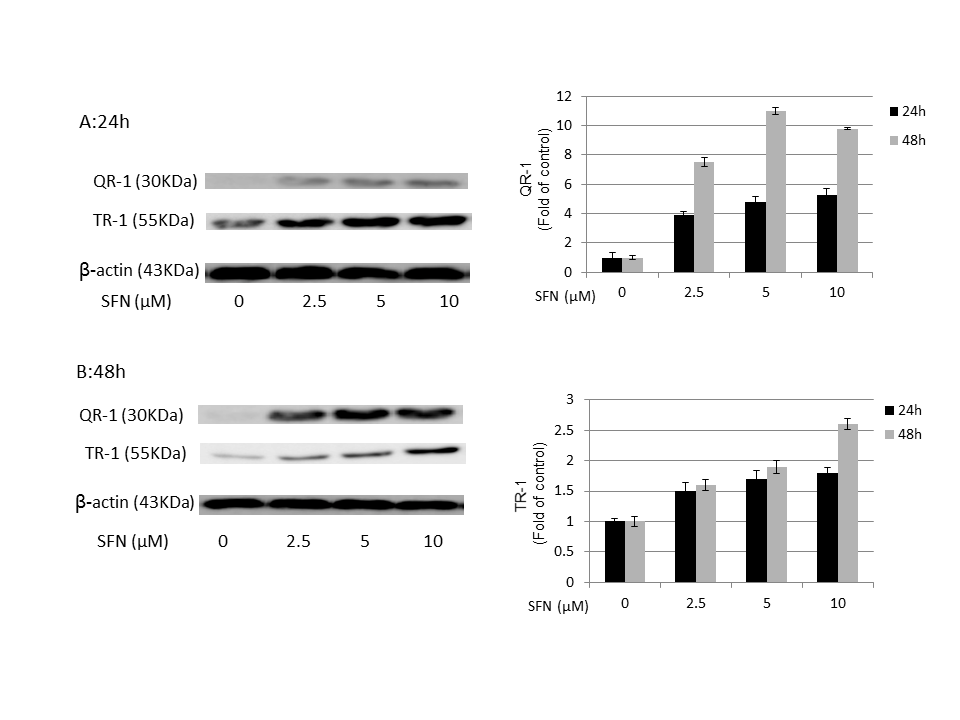

Supplement: S3 Fig — After seeding for 48 h, cells were exposed to SFN for 24 h (A) or 48 h (B). The expression of TR-1 and QR-1 were analysed by Western blot analysis. The band density was quantified using the Quantity One®. Data are the average of 3 experiments (±SD). (TIF) [file pone.0138771.s003.TIF]

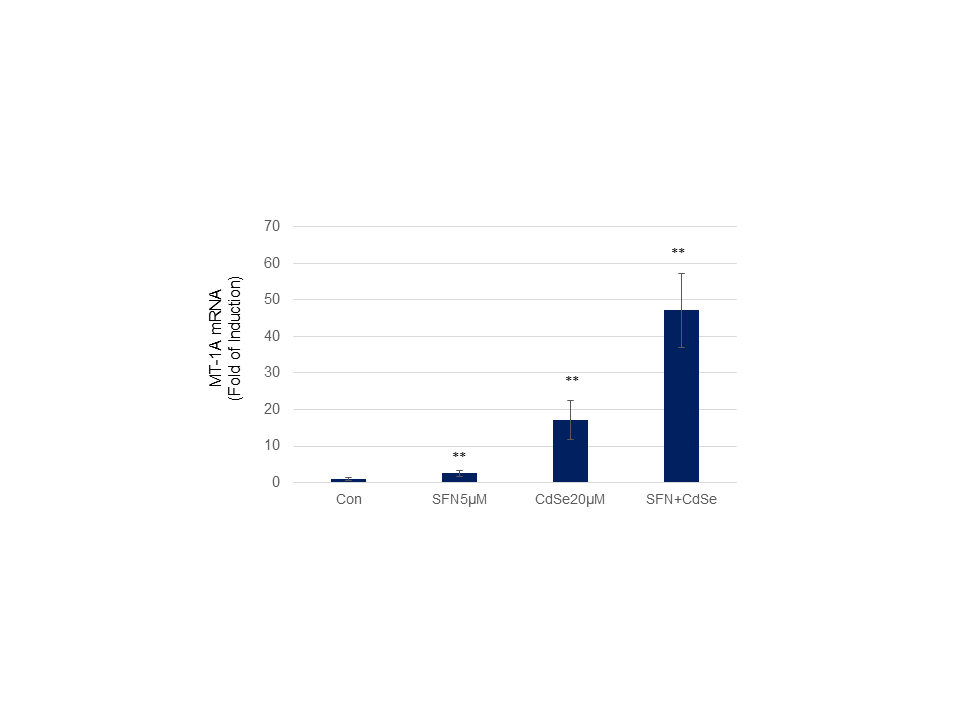

Supplement: S4 Fig — HHL-5 cells were treated with either SFN (5 μM), or CdSe (20 μM) and in their combination, i.e. pre-treatment SFN + CdSe for 24 h. DMSO (0.1%) as control. Total RNA was isolated using a GenElute™ total mammalian RNA kit (Sigma, UK). MT-1A mRNA was determined by TaqMan real-time PCR assays. The bar graphs represent means ± SD of three replicates. Statistical significance from the control, **p< 0.01. (TIF) [file pone.0138771.s004.TIF]

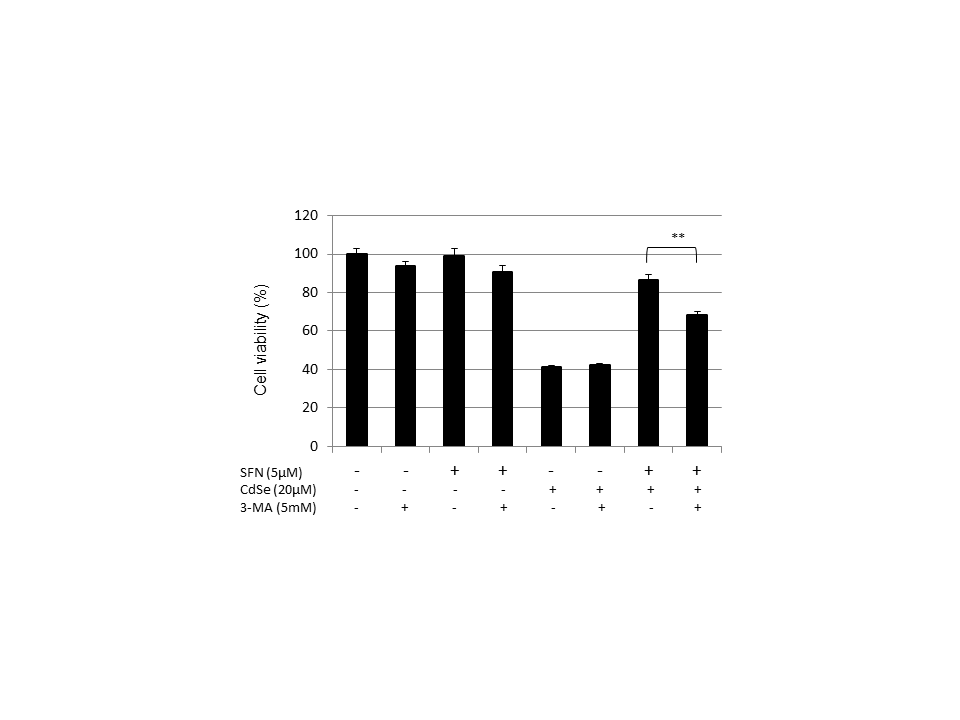

Supplement: S5 Fig — HHL-5 cells were pre-incubated with 3-MA (5 mM) for 6 h and then exposed to 5 μM SFN for 24 h. There was then a further 24 h exposure with 20 μM CdSe QDs. Cytotoxicity was measured by MTT assay. Data are shown as means ± SD (n = 6) (**P<0.01). (TIF) [file pone.0138771.s005.TIF]
